# Association between perfluoroalkyl and polyfluoroalkyl internal exposure and serum α-Klotho levels in middle-old aged participants

**DOI:** 10.3389/fpubh.2023.1136454

**Published:** 2023-05-04

**Authors:** Min Li, Yuanlin Ma, Wenli Cheng, Luyun Zhang, Cheng Zhou, Wenji Zhang, Wenjuan Zhang

**Affiliations:** ^1^Department of Public Health and Preventive Medicine, School of Medicine, Jinan University, Guangzhou, Guangdong, China; ^2^Guangdong Provincial Engineering and Technology Research Center for Tobacco Breeding and Comprehensive Utilization, Key Laboratory of Crop Genetic Improvement of Guangdong Province, Crops Research Institute, Guangdong Academy of Agricultural Sciences, Guangzhou, Guangdong, China; ^3^Reproductive Medicine Center, Guangdong Provincial Key Laboratory of Reproductive Medicine, The First Affiliated Hospital, Sun Yat-sen University, Guangzhou, China

**Keywords:** perfluoroalkyl and polyfluoroalkyl substances (PFAS), α-Klotho, NHANES, perfluorononanoic acid (PFNA), middle-aged women

## Abstract

**Purpose:**

Exposure to perfluoroalkyl and polyfluoroalkyl substances causes oxidative stress, which is strongly associated with adverse health effects. Klotho protein plays an anti-aging role via antioxidation activity.

**Methods:**

We investigated the levels of serum α-Klotho and PFAS exposure in adults who participated in the National Health and Nutrition Examination Survey from 2013 to 2016. A nationally representative subsample of 1,499 adults aged 40–79 years was analyzed for the associations of serum α-Klotho levels with serum PFAS exposures by correlation analysis and multiple general linear models. Of note, the potential confounding factors including age and gender were adjusted. Quantile-based g-computation models were used to assess the effects of mixed PFAS exposure on serum α-Klotho levels.

**Results:**

The weighted geometric mean of serum α-Klotho was 791.38 pg/mL for the subjects during 2013–2016. After adjusting for potential confounders, serum Klotho levels showed a statistically significant downward trend with increasing quartiles of PFOA and PFNA. Multivariate adjusted general linear regression analysis showed that increased exposure to PFNA was substantially associated with lower serum levels of α-Klotho, and each 1-unit increase in PFNA concentration was accompanied by a 20.23 pg/mL decrease in α-Klotho level; while no significant association was observed between other PFAS exposures and serum α-Klotho levels. It was negatively correlated between α-Klotho and Q4 for PFNA relative to the lowest quartile (Q1) of exposure (P = 0.025). It was found that the strongest negative correlation between PFNA exposure and serum α-Klotho levels was in the middle-aged (40–59 years) female participants. Furthermore, the mixture of the four PFAS substances showed an overall inverse association with serum α-Klotho concentrations, with PFNA being the major contributor.

**Conclusions:**

Taken together, in a representative sample of the U.S. middle-aged and elderly populations, serum concentrations of PFAS, especially PFNA, have been negatively associated with serum levels of α-Klotho, which is strongly associated with cognition and aging. It was important to note that the majority of associations were limited to middle-aged women. It will be meaningful to clarify the causal relationship and the pathogenic mechanisms of PFAS exposure and α-Klotho levels, which is helpful to aging and aging-related diseases.

## 1. Introduction

Perfluoroalkyl and polyfluoroalkyl substances (PFAS) are a critical group of persistent contaminants, has been used for decades in industrial and consumer applications mainly including protective coatings for carpets and clothing, water repellents, paper coatings, and surfactants (1). Their widespread use means that they are widely present in nature. PFAS-contaminated water and food, as well as inhalation of PFAS in an indoor setting, were the most common unavoidable exposure ways (2). Despite worldwide efforts were performed to control and reduce the use of PFAS, they are still detected in humans, wild animals, and various environmental matrices due to their ubiquitous application and stable carbon-fluorine bonds. Air pollution and cigarette smoke also contain high concentrations of PFOS. PFAS are environmental endocrine disruptors that can lead to serious human health problems including obesity, liver damage, kidney cancer, hypertension, immunotoxicity, etc. (3, 4). Specifically, it may induce cytotoxic effects through mechanisms such as increased oxidative stress (5–7), induction of apoptosis-related mitochondrial dysfunction (8, 9), and inhibition of gap junction intercellular communication (10). Some PFAS have the possibility of causing oxidative stress in human hepatocytes in terms of DNA damage and the production of reactive oxygen species (ROS), with genotoxic and cytotoxic potential (11). In addition, PFAS exposure is strongly associated with increased renal oxidative stress, and that a key pathway involving oxidative stress might be through disruptive effects on the peroxisome proliferator-activated receptor (PPAR) and its downstream functions (6). Several population studies have shown that serum PFAS concentrations are strongly associated with biomarkers of oxidative and nitrative stress. A general population cohort of Taiwan demonstrated that serum PFAS concentrations increased the levels of 8-nitroguanine and 8-hydroxy-2-deoxyguanosine (8-OHdG) (12). Similarly, 141 senior Koreans in a randomized controlled trial with elevated serum levels of perfluorododecanoic acid (PFDoDA) and PFOS concentrations also had higher levels of malondialdehyde and 8-OHdG in urine (13). *In vitro* and *in vivo* experiments, as well as population studies, have suggested that exposure to certain PFAS is associated with increased oxidative stress.

Klotho, initially considered to be a recognized suppressor gene of aging, has aroused great interest and enabled us to better understand the aging process (14). Klotho plays an important role as an anti-aging protein in the pathophysiology of multiple aging-related diseases, including atherosclerosis, diabetes, chronic kidney disease, neurological disorders, and cancers (15, 16). Klotho is a single transmembrane anti-aging protein in the form of α-Klotho, β-Klotho, and γ-Klotho, with different organ-specific expression and functions *in vivo* (17). Among these isoforms, α-Klotho, a transmembrane co-receptor for fibroblast growth factors 23 (FGF23) and a soluble factor in serum, is predominantly expressed in the kidney and brain, associated with phosphate metabolism, calcium uptake, energy metabolism, and various aging phenotypes (15, 18, 19).

We noticed that α-Klotho can inhibit oxidative stress. The α-Klotho stimulation increases the phosphorylation of forkhead box protein O3a (FOXO3a), inhibiting ROS-related oxidative stress. Transgenic mice overexpressing α-Klotho exhibited higher manganese superoxide dismutase (MnSOD) expression and lower oxidative stress, whereas α-Klotho deficiency significantly increased endogenous ROS production and oxidative stress aggravation (20, 21). Furthermore, overexpression of α-Klotho decreased H_2_O_2_-induced apoptosis, mitochondrial DNA breakage, β-galactosidase activity, superoxide anion production, and Bax protein expression (22). Low levels of ROS-associated stress can prolong cellular lifespan (23), and the anti-aging mechanism of α-Klotho may be related to ROS and its downstream signaling pathways.

PFAS may be pro-oxidants and alter oxidative stress levels. A recent study of NHANES data considering the critical role of renal failure first showed a modest association between PFAS exposure and low serum α-Klotho in healthy kidneys (24). In our current study, we again investigated the relationship between serum PFAS and serum α-Klotho levels in adults who participated in NHANES 2013–2016, in which adjustments were also made for potential confounders, and age- and sex-specific analyses were further conducted. Furthermore, we assessed the effects of mixed PFAS exposure on serum α-Klotho levels to understand their combined risk better. The relationship between PFAS and serum α-Klotho may remind us to prevent disorders associated with PFAS and serum α-Klotho, and α-Klotho may be a preventative and therapeutic target for its related diseases after exposure to PFAS.

## 2. Materials and methods

### 2.1. Study population

NHANES is conducted by the National Center for Health Statistics (NCHS) of the Centers for Disease Control and Prevention (CDC) as a major program to assess the health and nutritional status of the adults and children in the United States, with a deliberately sampled population intended to be representative of the US population by age, ethnicity, and other demographic factors. The survey is unique, combining interviews and physical examinations, and is publicly available at www.cdc.gov/nchs/nhanes/, with the detailed procedures previously described (25). The NCHS Research Ethics Review Board examined and approved the NHANES protocol.

**Figure 1:**
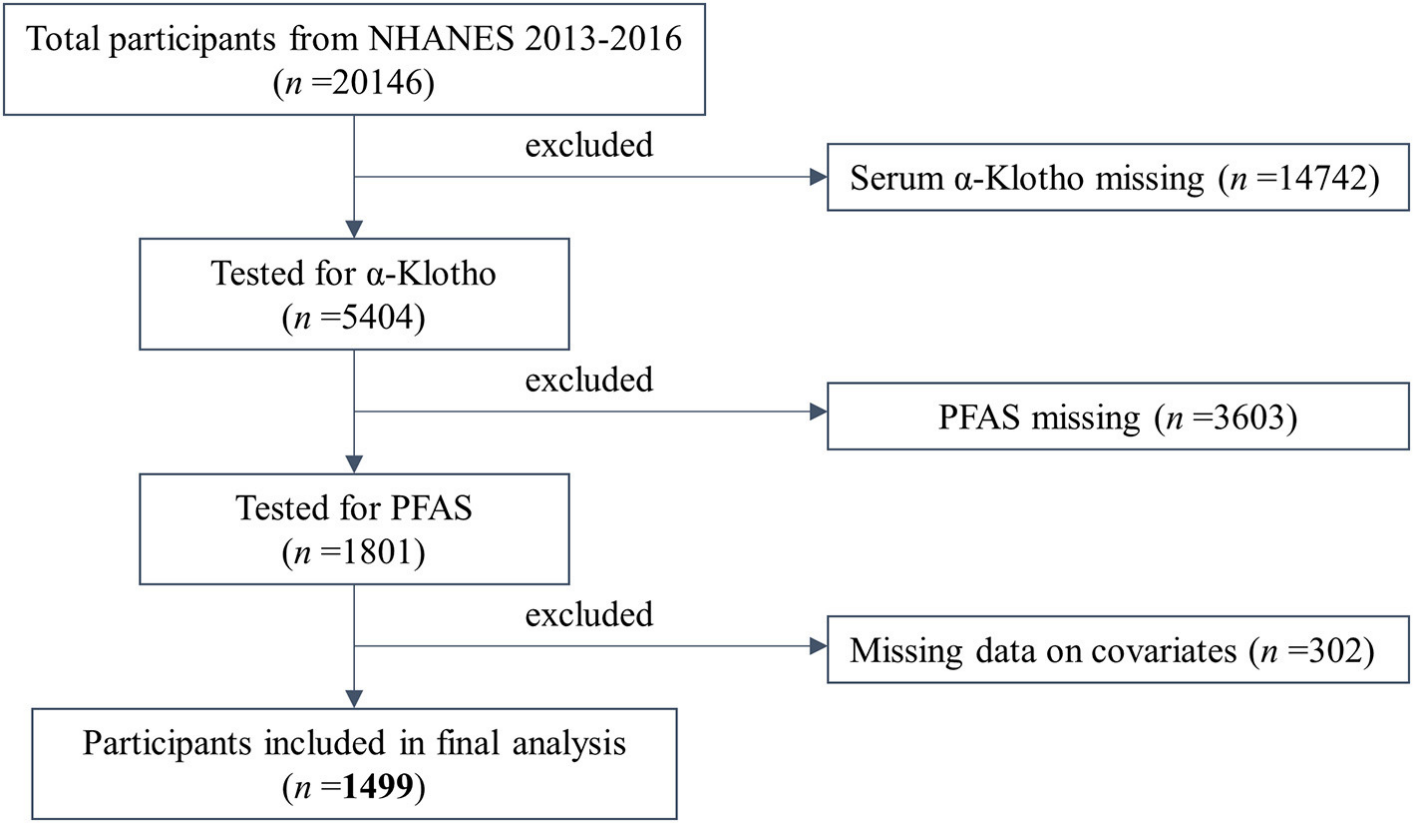
Flow chart of the population included in the final analysis of this study. *n* = 1,499, NHANES, USA, 2013–2016.

Participants in this study were drawn from the NHANES cycles for 2013–2014 and 2015–2016. Due to the availability of α-Klotho data, we only included adult participants aged from 40 to 79 years. As shown in Figure 1, the sample size was reduced to 1,499 after eliminating participants with missing data on serum α-Klotho (*n* = 14,742), PFAS (*n* = 3,603), and any missing covariates included in the study (*n* = 302).

### 2.2. Measurement of serum α-Klotho level

Fresh frozen serum samples, stored at −80°C, were used for α-Klotho concentration measurements by a CDC certified laboratory using a commercially available ELISA kit (IBL International, Japan). The IBL ELISA method was extensively validated to measure Klotho concentrations in human samples prior to formal analysis. The sensitivity of the assay was 6.15 pg/mL. For quality assurance, the average of two replicate analyses was used, and the detailed description of the analytical method was included as previous document (26). All measurements were completed by the U.S. Centers for Disease Control and Prevention for the ongoing NHANES.

### 2.3. Measurements of PFAS

One third of the population aged 12 years and older was randomly selected in each survey cycle and examined for the presence of PFAS in their serum by using online solid-phase extraction-high performance liquid chromatography-turbine ionization-tandem mass spectrometry measurements. A total of 12 PFAS were detected from 2013 to 2016. Details of the analytical method could be found on the NHANES website (27–29).

This study focused on those PFAS compounds with detection rates higher than 80% among survey participants from 2013 to 2016, including PFOA, PFOS, PFNA, and PFHxS. The concentrations of linear perfluorooctanoate (n-PFOA) and branched isomers of perfluorooctanoate (Sb-PFOA) were summed to total PFOA. The concentrations of linear perfluorooctane sulfonate (n-PFOS) and monomethyl branched isomers of PFOS (Sm-PFOS) were calculated as total PFOS. The values below the lower limit of detection (LLOD) were imputed by the LLOD divided by the square root of 2 (30).

### 2.4. Covariates

Based on clinical significance and univariate analysis, covariates were specifically selected from questionnaire data on demographics, socioeconomic status, health behaviors, and health status (31). We included age, gender, race, household income, education, physical activity, body mass index (BMI), smoking, alcohol consumption, diabetes, hypertension, urine albumin/urine creatinine (uACR), and glomerular filtration rate (eGFR) as covariates.

Sociodemographic variables included age (40–59 and 60–79), gender (male and female), race (non-Hispanic white, non-Hispanic black, Mexican American, and other race), family income to poverty ratio (<1 and ≥1), and education (less than high school, high school or equivalent, and more than high school). Health-related behavior information included physical activity (yes and no), BMI (<25 kg/m^2^, 25 to < 30 kg/m^2^, and ≥30 kg/m^2^), smoking status (never smoker, past smoker, and current smoker), and alcohol consumption (yes and no). Health status included a history of hypertension (yes and no), and a history of diabetes (yes and no). Of these, physical activity was classified as moderate or vigorous physical recreational activity vs. no activity. BMI was calculated as weight in kilograms divided by height in meters squared. For smoking status, past smokers and never smokers were categorized according to having smoked at least 100 cigarettes in their lifetime. Those who smoked more than 100 cigarettes during their lifetime and smoked some days or every day were defined as current smokers. Alcohol consumers and non-alcohol consumers were separated based on having consumed at least 12 alcoholic beverages in their lifetime.

Estimated glomerular filtration rate (eGFR) was calculated according to the CKD-EPI-Scr creatinine for participants aged ≤70 years (32), and the BIS1-Scr creatinine equation for participants aged >70 years (33). Urinary albumin and creatinine levels were gold-scaled and calibrated according to the NHANES's recommended standard method, where uACR = urinary albumin/urine creatinine.

### 2.5. Statistical analyses

In this study, we took into account the complex survey design and data clustering, following the NHANES procedure of using subsample population weights to obtain estimates typical of the U.S. population. Since the data for the four serum PFAS and α-Klotho concentrations were significantly right-skewed, the data were log-transformed to obtain a near-normal distribution in part of our analysis (34, 35). Weighted geometric means and 95% CIs of serum α-Klotho levels and the four serum PFAS were calculated for the entire study population as well as for covariates based on the characteristics of the study population. To determine the statistical significance of the mean differences, a *t*-test was used for two-group comparisons, and a one-way ANOVA was conducted for comparisons of multiple groups.

PFAS levels were analyzed as continuous variables and quartiles, respectively. A Pearson correlation analysis on the log-transformed serum α-Klotho levels and these four log-transformed PFAS concentrations was performed. The adjusted geometric mean of serum α-Klotho was calculated by quartiles of serum PFAS levels. The median value of the quartiles was applied to the PFAS exposure variable, as a continuous variable in a linear trend test. The relationship between serum α-Klotho (dependent variable) and serum PFAS (independent variable) was evaluated using linear regression, and the corresponding beta coefficients and 95% CIs were calculated. Beta coefficients and 95% CIs for serum α-Klotho levels among individuals with higher levels of serum PFAS (quartiles 2 through 4) compared with those with the lowest level of serum PFAS (quartile 1) were also calculated. We further performed regression analyses stratified by age and sex to ascertain an age- or gender-specific association between PFAS concentrations and serum α-Klotho levels. The above analysis was performed using the package “nhanesR” version 0.9.4.0. Using the R package “qgcomp,” the quantile-based g-computation models were used to assess the relationship between combined exposures to PFAS and serum α-Klotho levels. All statistical analyses were performed using R version 4.2.1 and Microsoft Excel 2016. *P* < 0.05 was considered statistically significant.

## 3. Results

### 3.1. General characteristics of the study participants

The weighted population characteristics of the study participants were presented in Table 1. The weighted mean age was 56.72 years, in the range of 40–79 years, with more females (53.50%). The constituent ratio of the study population was 39.76% for non-Hispanic white, 20.81% for non-Hispanic black, 15.01% for Mexican American, and 24.42% for other races. Most of the participants had a family income above the poverty level (79.99%) and had more than a high school diploma (54.90%). The weighted mean BMI was 29.71 kg/m^2^ with a high number of overweight and obese individuals (33.62 and 43.63%, respectively). The majority of participants engaged in physical activities (71.58%), never smoked (53.17%), drank alcohol (85.46%), did not have diabetes (71.65%), and had hypertension (54.57%).

**Table 1:** Weighted characteristics of the study participants in the 2013–2016 NHANES (*n* = 1,499).

**Characteristic**	***n*** **(%)^a^**
**Age (year)**	
40–59	806 (53.77)
60–79	693 (46.23)
**Sex**	
Male	697 (46.50)
Female	802 (53.50)
**Race**	
Non-Hispanic White	596 (39.76)
Non-Hispanic Black	312 (20.81)
Mexican American	225 (15.01)
Other race	366 (24.42)
**Family income to poverty ratio**	
Below poverty (< 1)	300 (20.01)
Above poverty (≥1)	1,199 (79.99)
**Education**	
Less than high school	340 (22.68)
High school or equivalent	336 (22.41)
More than high school	823 (54.90)
**Physical activity**	
Yes	1,073 (71.58)
No	426 (28.42)
**BMI (kg/m** ^2^ **)**	
< 25	341 (22.75)
25 to < 30	504 (33.62)
≥30	654 (43.63)
**Smoking status**	
Never smoker	797 (53.17)
Past smoker	417 (27.82)
Current smoking	285 (19.01)
**Alcohol consumption**	
Yes	1,281 (85.46)
No	218 (14.54)
**Diabetes**	
Yes	425 (28.35)
No	1,074 (71.65)
**Hypertension**	
Yes	818 (54.57)
No	618 (45.43)
**NHANES survey cycle**	
2013–2014	791 (52.77)
2015–2016	708 (47.23)

a Unweighted *n* and weighted %.

Weighted geometric means (GM) and 95% CIs of serum α-Klotho and serum PFOA, PFOS, PFNA, and PFHxS levels were calculated for the entire study population as well as for covariates based on the characteristics of the study population separately in Table 2. For the entire study population, the geometric mean (95% CI) α-Klotho level was 791.38 (776.17, 806.88) pg/mL. Serum α-Klotho levels varied significantly by age, sex, race, education, BMI, smoking status, alcohol consumption, and hypertension history. As expected, the geometric mean serum α-Klotho level was significantly higher in middle adults aged from 40 to 59 years (814.93 pg/mL) than that in older participants aged from 60 to 79 years (756.82 pg/mL). The higher α-Klotho levels were also found in female (male vs. female, 773.81 vs. 808.30 pg/mL), black (non-Hispanic white, non-Hispanic black, Mexican American, and other race; 784.26, 833.33, 783.78, and 807.59 pg/mL), and highly educated (less than high school, high school or equivalent, and more than high school; 774.98, 753.20, and 807.68 pg/mL) subjects. Furthermore, α-Klotho serum levels were significantly lower in overweight (777.00 pg/mL) and obese (781.65 pg/mL) people than in normal and low weight people (830.01 pg/mL). For health-related factors, those with higher serum α-Klotho levels never smoked (current smoker, past smoker, and never smoker; 719.62, 797.65, and 815.98 pg/mL), did not drink alcohol (drinker vs. non-drinker; 783.50 vs. 875.76 pg/mL), and were never diagnosed with hypertension (yes vs. no; 772.09 vs. 810.22 pg/mL).

**Table 2:** Serum levels of four PFAS and α-Klotho in pooled 2013–2016 NHANES (*n* = 1,499).

**Characteristic**	**Serum α-Klotho^a^ (pg/mL)**	***P***	**Serum PFOA^a^ (ng/mL)**	***P***	**Serum PFOS^a^ (ng/mL)**	***P***	**Serum PFNA^a^ (ng/mL)**	***P***	**Serum PFHxS^a^ (ng/mL)**	***P***
**Age (year)**										
40–59	814.93 (795.30, 835.04)	**0.012**	1.83 (1.64, 2.04)	**0.020**	5.16 (4.72, 5.64)	**< 0.001**	0.65 (0.59, 0.71)	**0.002**	1.22 (1.08, 1.39)	**0.002**
60–79	756.82 (728.39, 786.35)		2.29 (2.11, 2.49)		7.34 (6.60, 8.16)		0.83 (0.76, 0.91)		1.76 (1.61, 1.93)	
**Sex**										
Male	773.81 (751.37, 796.93)	**0.011**	2.21 (2.05, 2.38)	**< 0.001**	7.64 (6.99, 8.36)	**< 0.001**	0.76 (0.69, 0.84)	**0.007**	1.90 (1.74, 2.06)	**< 0.001**
Female	808.30 (788.55, 828.54)		1.82 (1.67, 1.98)		4.67 (4.33, 5.04)		0.67 (0.62, 0.73)		1.07 (0.97, 1.19)	
**Race**										
Non-Hispanic White	784.26 (765.52, 803.47)	**0.040**	2.14 (1.96, 2.35)	**0.006**	5.92 (5.47, 6.41)	**< 0.001**	0.70 (0.64, 0.77)	**0.007**	1.50 (1.34, 1.67)	**0.007**
Non-Hispanic Black	833.33 (794.21, 874.39)		1.77 (1.60, 1.96)		7.18 (5.88, 8.78)		0.81 (0.71, 0.91)		1.27 (1.09, 1.48)	
Mexican American	783.78 (753.77, 814.99)		1.43 (1.30, 1.58)		4.47 (4.07, 4.90)		0.62 (0.56, 0.68)		1.13 (1.02, 1.25)	
Other race	807.59 (773.89, 842.75)		1.73 (1.60, 1.86)		6.02 (5.28, 6.86)		0.78 (0.70, 0.86)		1.23 (1.05, 1.45)	
**Family income to poverty ratio**										
Below poverty (< 1)	771.01 (740.29, 803.02)	0.176	1.75 (1.45, 2.11)	**0.002**	5.36 (4.64, 6.20)	0.600	0.64 (0.56, 0.73)	**0.006**	1.43 (1.13, 1.80)	0.428
Above poverty (≥1)	794.20 (778.35, 810.37)		2.04 (1.91, 2.18)		6.01 (5.58, 6.47)		0.73 (0.67, 0.78)		1.41 (1.29, 1.54)	
**Education**										
Less than high school	774.98 (739.04, 812.67)	**0.013**	1.83 (1.55, 2.17)	**0.016**	6.06 (5.31, 6.92)	0.338	0.70 (0.62, 0.79)	0.480	1.46 (1.23, 1.74)	0.615
High school or equivalent	753.20 (720.10, 787.81)		2.07 (1.75, 2.44)		6.01 (5.05, 7.15)		0.73 (0.63, 0.85)		1.38 (1.15, 1.65)	
More than high school	807.68 (787.16, 828.73)		2.01 (1.87, 2.17)		5.88 (5.36, 6.45)		0.71 (0.65, 0.78)		1.42 (1.26, 1.59)	
**Physical activity**										
Yes	795.54 (772.39, 819.40)	0.389	2.04 (1.90, 2.19)	0.195	5.89 (5.46, 6.35)	0.222	0.72 (0.67, 0.78)	0.345	1.42 (1.29, 1.56)	0.902
No	778.35 (747.44, 810.55)		1.88 (1.69, 2.08)		6.06 (5.32, 6.92)		0.69 (0.62, 0.77)		1.40 (1.24, 1.57)	
**BMI (kg/m** ^2^ **)**										
< 25	830.01 (797.47, 863.88)	**0.013**	2.05 (1.81, 2.31)	0.300	6.04 (5.25, 6.95)	0.223	0.75 (0.67, 0.84)	**0.009**	1.34 (1.15, 1.55)	**0.036**
25 to < 30	777.00 (747.51, 807.64)		2.13 (1.92, 2.35)		6.34 (5.76, 6.98)		0.76 (0.68, 0.85)		1.56 (1.39, 1.75)	
≥30	781.65 (761.02, 802.85)		1.88 (1.74, 2.04)		5.57 (5.04, 6.14)		0.66 (0.60, 0.73)		1.35 (1.22, 1.50)	
**Smoking status**										
Never smoker	815.98 (795.70, 836.77)	**< 0.001**	1.98 (1.86, 2.10)	0.682	6.04 (5.55, 6.58)	**0.003**	0.73 (0.67, 0.80)	0.210	1.36 (1.22, 1.50)	0.556
Past smoker	797.65 (766.01, 830.61)		2.04 (1.83, 2.28)		6.42 (5.79, 7.10)		0.72 (0.64, 0.81)		1.57 (1.43, 1.74)	
Current smoking	719.62 (688.47, 752.18)		2.00 (1.63, 2.46)		5.01 (4.32, 5.79)		0.66 (0.58, 0.76)		1.34 (1.05, 1.72)	
**Alcohol consumption**										
Yes	783.50 (769.55, 797.71)	**0.007**	2.02 (1.88, 2.17)	0.085	5.93 (5.49, 6.41)	0.251	0.71 (0.66, 0.77)	0.697	1.43 (1.31, 1.57)	0.327
No	875.76 (803.82, 954.13)		1.84 (1.65, 2.05)		5.92 (5.06, 6.93)		0.71 (0.62, 0.81)		1.26 (1.08, 1.46)	
**Diabetes**										
Yes	799.49 (770.01, 830.10)	0.321	1.78 (1.60, 1.98)	0.132	5.87 (5.01, 6.88)	0.348	0.67 (0.58, 0.77)	0.274	1.36 (1.20, 1.54)	0.872
No	789.17 (772.48, 806.23)		2.07 (1.90, 2.24)		5.95 (5.54, 6.38)		0.73 (0.67, 0.79)		1.43 (1.30, 1.57)	
**Hypertension**										
Yes	772.09 (753.07, 791.59)	**0.017**	2.03 (1.92, 2.15)	0.897	6.22 (5.58, 6.94)	**0.001**	0.73 (0.67, 0.79)	0.122	1.44 (1.31, 1.58)	0.354
No	810.22 (785.22, 836.02)		1.97 (1.78, 2.19)		5.67 (5.29, 6.07)		0.70 (0.64, 0.76)		1.39 (1.25, 1.55)	

Bold font indicates a statistically significant difference.

a Presented as weighted geometric mean (GM, 95% CI).

The geometric mean (95% CI) of serum PFOA, PFOS, PFNA, and PFHxS for the total study population were 2.00 (1.87–2.14) ng/mL, 5.93 (5.50–6.39) ng/mL, 0.71 (0.66–0.77) ng/mL, and 1.41 (1.29–1.54) ng/mL, respectively. Serum concentrations of these four PFAS were higher in older adults (60–79 years) than those in the middle (40–59 years), with higher blood concentrations in males than those in females. Four PFAS levels were found to be lower in the Mexican American population significantly. Participants with high family income had higher concentrations of PFOA and PFNA in their blood with significance. PFNA and PFHxS concentrations were higher in the blood of overweight participants and PFOS in the blood of hypertensive participants. Interestingly, blood PFOS concentrations were higher in participants who were past smokers or never smokers compared with those in current smokers.

### 3.2. Measurements and correlations of serum PFAS and the relationships with α-Klotho

The PFAS concentrations in the study population were shown in Supplementary Table 1. All four PFAS were detectable in more than 98% of the population. PFOS had the highest detection rate as 99.40%, followed by PFOA as 99.33%, PFNA as 98.87% and PFHxS as 98.73%. Notably, PFOS had the highest concentration with GM 5.93 ng/mL, while PFNA had the lowest with GM 0.71 ng/mL.

**Figure 2:**
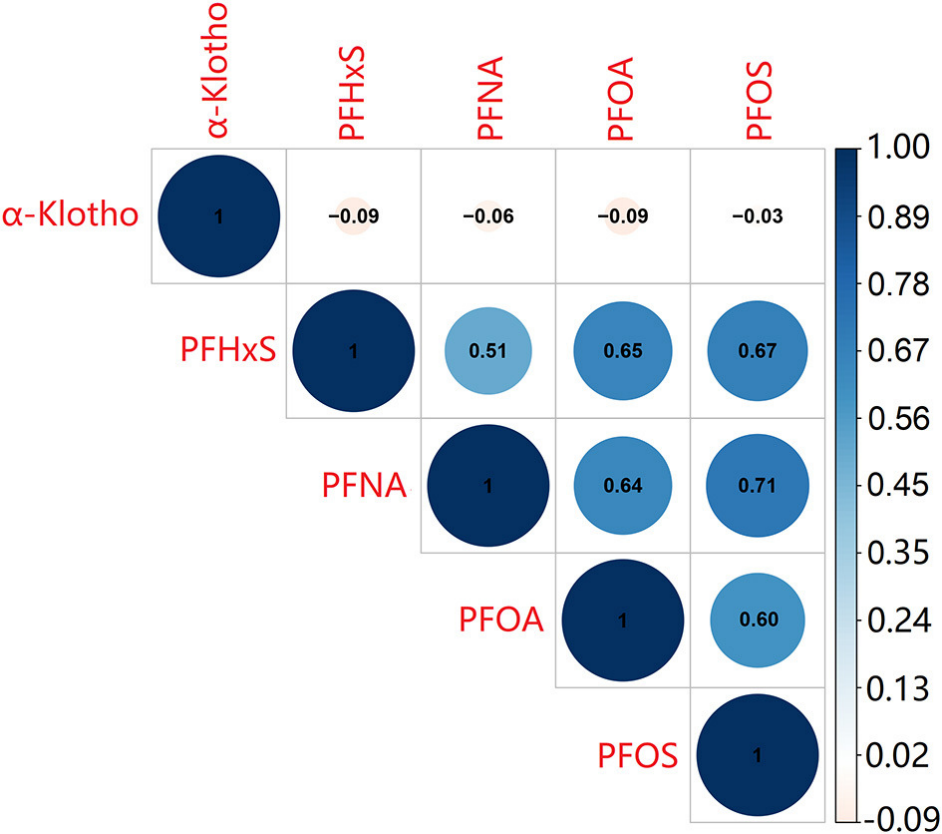
The correlation between the four PFAS and α-Klotho levels. The test was Pearson's correlation coefficients among log-transformed indices.

From Figure 2, it showed the correlation matrix of log-transformed α-Klotho and serum PFAS concentrations. Serum α-Klotho levels presented a negative correlation with blood PFHxS, PFNA, and PFOA levels separately. The correlation coefficients ranged from −0.09 to −0.06, with greater correlation coefficients for PFHxS and PFNA levels compared with PFOA. However, serum PFOS levels had no significant correlation with serum α-Klotho. Furthermore, the correlation coefficients among the four PFAS were significance (*P* < 0.001). The strongest correlation was found between PFOS and PFNA (*r* = 0.71), followed by the correlation between PFOS and PFHxS (*r* = 0.67), while the weakest correlation was between PFNA and PFHxS (*r* = 0.51).

### 3.3. Mean serum α-Klotho levels by quartiles of PFAS

**Figure 3:**
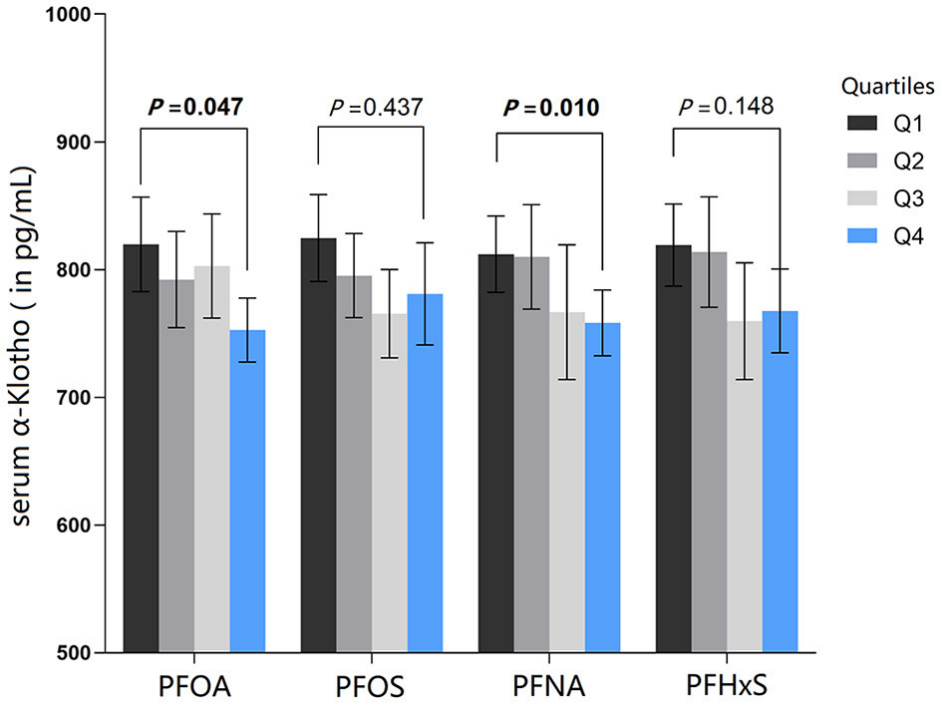
All-adjusted geometric means (and 95% CIs) of serum α-Klotho by quartiles of serum PFAS. The all-adjusted model for serum PFAS were adjusted for age, gender, race, family income to poverty ratio, education, physical activity, BMI, smoking, alcohol consumption, diabetes, hypertension, uACR, and eGFR.

As shown from Figure 3 and Table 3, the geometric mean serum α-Klotho levels revealed a statistically significant declining trend with rising serum PFOA (*P* = 0.007), PFNA (*P* = 0.005) and PFHxS (*P* = 0.007). The adjusted geometric means of serum α-Klotho showed a statistically significant downward trend with serum PFOA (*P* = 0.047) and PFNA (*P* = 0.010), after adjusting for all covariables.

**Table 3:** Multivariate adjusted geometric means and 95% CI of serum α-Klotho by quartiles of PFAS.

	***n***	**Geometric mean of serum α- Klotho in pg/mL (95% CI)**	**Unadjusted model**	**Adjusted model^a^**
			***P*** **for trend**^b^	***P*** **for trend**^b^
**By serum PFOA quartile**				
Q1	387	819.82 (784.42, 856.82)	**0.007**	**0.047**
Q2	375	792.26 (756.27, 829.95)		
Q3	365	802.86 (764.13, 843.56)		
Q4	372	752.63 (728.36, 777.72)		
**By serum PFOS quartile**				
Q1	381	824.79 (792.09, 858.84)	0.251	0.437
Q2	367	795.33 (763.73, 828.25)		
Q3	375	765.57 (732.42, 800.22)		
Q4	376	781.04 (743.00, 821.02)		
**By serum PFNA quartile**				
Q1	465	812.16 (783.39, 841.98)	**0.005**	**0.010**
Q2	390	810.01 (771.05, 850.94)		
Q3	302	766.76 (717.39, 819.54)		
Q4	342	758.33 (733.38, 784.14)		
**By serum PFHxS quartile**				
Q1	427	819.29 (788.41, 851.38)	**0.007**	0.148
Q2	357	813.83 (772.79, 857.04)		
Q3	340	759.69 (716.56, 805.42)		
Q4	375	767.67 (736.20, 800.48)		

Bold font indicates a statistically significant difference.

a Adjusted models for PFAS were adjusted for age, gender, race, family income to poverty ratio, education, physical exercise, BMI, smoking, drinking status, diabetes, hypertension, uACR, and eGFR.

b Test for trend based on variable containing median value for each quartile.

### 3.4. Linear relationships between serum α-Klotho and exposure to PFAS

Multiple general linear models were then performed to investigate the association between blood PFAS concentrations and serum α-Klotho levels in the total population. Associations between continuous and quartiles of PFAS and α-Klotho measurement were reported in Table 4. In most cases, continuous PFAS concentrations and α-Klotho measurements were negatively correlated, but only the association between PFNA and α-Klotho was statistically significant in both unadjusted and adjusted models. After adjusting for all covariates, α-Klotho levels were decreased by 20.23 pg/mL for each 1-unit increase in PFNA concentration (*P* = 0.036). In quartile analysis, there was a significant negative association between α-Klotho and Q4 of PFOA [unadjusted β (95% CI) = −79.41 (−138.20, −20.63), *P* = 0.010], Q3 of PFOS [unadjusted β (95% CI) = −66.64 (−125.70, −7.59), *P* = 0.028], Q4 of PFNA [unadjusted β (95% CI) = −57.83 (−104.03, −11.64), *P* = 0.016], and Q4 of PFHxS [unadjusted β (95% CI) = −60.55 (−111.34, −9.75), *P* = 0.021] in the unadjusted model relative to the lowest quartile (Q1) of each exposure. However, in the adjusted model, only a significant negative correlation was found between α-Klotho and Q4 of PFNA [adjusted β (95% CI) = −61.36 (−113.26, −9.45), *P* = 0.025] relative to Q1.

**Table 4:** Linear regression analysis of serum α-Klotho levels and serum PFAS concentrations among study participants.

**Biomarkers**	**Model**	**By serum PFAS**		**By quartile of serum PFAS**		
		β **(95% CI)**	***P***	**Quartiles**	β **(95% CI)**	***P***
PFOA	Unadjusted	−3.70 (−8.28, 0.88)	0.109	Q1	Reference	
				Q2	−36.88 (−106.76, 32.99)	0.288
				Q3	−13.58 (−87.29, 60.13)	0.708
				Q4	−79.41 (−138.20, −20.63)	**0.010**
	Adjusted^a^	−1.95 (−5.42, 1.52)	0.245	Q1	Reference	
				Q2	−39.56 (−111.29, 32.17)	0.250
				Q3	−3.39 (−80.91, 74.14)	0.925
				Q4	−61.46 (−124.43, 1.51)	0.055
PFOS	Unadjusted	−0.75 (−2.77, 1.26)	0.475	Q1	Reference	
				Q2	−29.16 (−90.72,32.41)	0.340
				Q3	−66.64 (−125.70, −7.59)	**0.028**
				Q4	−37.55 (−100.46, 25.36)	0.231
	Adjusted^a^	−0.13 (−2.15, 1.89)	0.889	Q1	Reference	
				Q2	−20.56 (−89.60, 48.49)	0.526
				Q3	−64.19 (−130.20, 1.83)	0.056
				Q4	−25.54 (−94.33, 43.25)	0.431
PFNA	Unadjusted	−22.36 (−37.88, −6.84)	**0.006**	Q1	Reference	
				Q2	−2.75 (−70.76, 65.25)	0.934
				Q3	−39.47 (−103.69, 24.75)	0.218
				Q4	−57.83 (−104.03, −11.64)	**0.016**
	Adjusted^a^	−20.23 (−38.87, −1.58)	**0.036**	Q1	Reference	
				Q2	−2.62 (−66.00, 60.75)	0.929
				Q3	−40.36 (−101.39, 20.66)	0.173
				Q4	−61.36 (−113.26, −9.45)	**0.025**
PFHxS	Unadjusted	−13.97 (−22.91, −5.03)	**0.003**	Q1	Reference	
				Q2	−9.47 (−80.21, 61.28)	0.786
				Q3	−57.71 (−129.59, 14.16)	0.111
				Q4	−60.55 (−111.34, −9.75)	**0.021**
	Adjusted^a^	−8.09 (−18.50, 2.32)	0.116	Q1	Reference	
				Q2	−2.95 (−75.77, 69.86)	0.930
				Q3	−37.16 (−109.63, 35.32)	0.283
				Q4	−36.20 (−96.39, 24.00)	0.213

Bold font indicates a statistically significant difference.

a Adjusted models for PFAS were adjusted for age, gender, race, family income to poverty ratio, education, physical exercise, BMI, smoking, drinking status, diabetes, hypertension, uACR, and eGFR.

### 3.5. Stratified analysis by age and gender

Then the relationships between serum α-Klotho and PFAS concentrations were stratified by age and gender as shown in Table 5. After stratified by age, only a significantly negative association between PFNA concentration and α-Klotho was observed in middle adults (40–59 years) in the adjusted model with a β of −50.98 (*P* = 0.017). In sex-stratified analyses, a significantly negative association between the concentration of PFNA and α-Klotho was observed only in the females in the adjusted model with a β of −37.01 (*P* = 0.047). Additionally, when stratified by age and sex, significant negative associations of PFOA, PFNA, and PFHxS with serum α-Klotho were found in the unadjusted model in middle (40–59 years) female participants. A negative correlation between PFNA and serum α-Klotho was also found in older (60–79 years) females. However, in the adjusted model, we only found a negative association of PFNA with serum α-Klotho in middle-aged (40–59 years) female participants, with an adjusted β of −109.66 (*P* < 0.001).

**Table 5:** Linear regression analysis of serum α-Klotho levels and serum PFAS concentrations among study participants stratified by age and gender.

**Biomarkers**	**Model**	**Stratification by age**			**Stratification by gender**			**Stratification by age and gender**		
		**Age**	β **(95% CI)**	***P***	**Gender**	β **(95% CI)**	***P***	**Group**	β **(95% CI)**	***P***
PFOA	Unadjusted	40–59	−2.29 (−4.75, 0.17)	0.067	Male	−8.28 (−22.55, 6.00)	0.246	Males 40–59	−0.02 (−22.08, 22.05)	0.999
		60–79	**−7.57 (−14.06**, **−1.08)**	**0.024**	Female	−3.29 (−7.10, 0.51)	0.087	Males 60–79	−11.08 (−28.00, 5.85)	0.190
								Females 40–59	**−2.36 (−4.48**, **−0.25)**	**0.030**
								Females 60–79	−6.37 (−13.06, 0.32)	0.061
	Adjusted^a^	40–59	−1.00 (−3.36, 1.37)	0.380	Male	−3.58 (−16.81, 9.65)	0.569	Males 40–59	8.64 (−15.51, 32.77)	0.456
		60–79	−5.78 (−13.18, 1.62)	0.115	Female	−1.91 (−5.36, 1.55)	0.254	Males 60–79	−10.95 (−27.17, 5.27)	0.167
								Females 40–59	−1.35 (−4.13, 1.44)	0.318
								Females 60–79	−3.73 (−11.20, 3.75)	0.303
PFOS	Unadjusted	40–59	−0.74 (−3.29, 1.81)	0.557	Male	0.55 (−2.04, 3.15)	0.666	Males 40–59	0.58 (−3.01, 4.16)	0.744
		60–79	0.77 (−2.26, 3.79)	0.609	Female	−1.40 (−4.28, 1.48)	0.327	Males 60–79	1.70 (−2.87, 6.28)	0.452
								Females 40–59	−1.21 (−6.01, 3.59)	0.609
								Females 60–79	0.57 (−2.30, 3.43)	0.689
	Adjusted^a^	40–59	−0.72 (−3.44, 2.01)	0.580	Male	1.16 (−2.01, 4.33)	0.445	Males 40–59	1.08 (−3.07, 5.23)	0.585
		60–79	0.85 (−2.56, 4.27)	0.599	Female	−2.19 (−5.34, 0.97)	0.158	Males 60–79	1.17 (−4.92, 7.26)	0.683
								Females 40–59	−3.47 (−8.05, 1.12)	0.127
								Females 60–79	−0.18 (−3.31, 2.96)	0.906
PFNA	Unadjusted	40–59	**−39.57 (−70.25**, **−8.89)**	**0.013**	Male	0.83 (−31.08, 32.73)	0.958	Males 40–59	−0.43 (−56.07, 55.21)	0.987
		60–79	−5.99 (−20.04, 8.07)	0.391	Female	**−31.48 (−56.68**, **−6.29)**	**0.016**	Males 60–79	14.60 (−30.52, 59.71)	0.512
								Females 40–59	**−69.77 (−123.10**, **−16.44)**	**0.012**
								Females 60–79	**−11.81 (−22.79**, **−0.82)**	**0.036**
	Adjusted^a^	40–59	**−50.98 (−91.07**, **−10.88)**	**0.017**	Male	14.11 (−19.89, 48.11)	0.386	Males 40–59	10.59 (−53.02, 74.19)	0.726
		60–79	−1.96 (−18.11, 14.19)	0.797	Female	**−37.01 (−73.41**, **−0.62)**	**0.047**	Males 60–79	17.424 (−33.30, 68.15)	0.469
								Females 40–59	**−109.66 (−163.79**, **−55.52)**	**< 0.001**
								Females 60–79	−8.17 (−24.21, 7.88)	0.293
PFHxS	Unadjusted	40–59	**−15.05 (−28.96**, **−1.14)**	**0.035**	Male	−5.17 (−18.27, 7.93)	0.426	Males 40–59	−6.41 (−26.85, 14.02)	0.526
		60–79	−8.27 (−17.41, 0.86)	0.074	Female	**−19.05 (−30.39**, **−7.72)**	**0.002**	Males 60–79	−1.07 (−17.47, 15.34)	0.895
								Females 40–59	**−19.29 (−38.39**, **−0.18)**	**0.048**
								Females 60–79	−12.42 (−25.39, 0.55)	0.060
	Adjusted^a^	40–59	−7.26 (−24.24, 9.72)	0.373	Male	−0.88 (−15.87, 14.12)	0.902	Males 40–59	−1.29 (−24.31, 21.73)	0.906
		60–79	−6.67 (−17.39, 4.04)	0.202	Female	−13.52 (−27.13, 0.10)	0.052	Males 60–79	0.55 (−15.99, 17.09)	0.943
								Females 40–59	−13.60 (−36.89, 9.69)	0.231
								Females 60–79	−12.25 (−26.89, 2.39)	0.094

Bold font indicates a statistically significant difference.

a Adjusted models for PFAS were adjusted for age, gender, race, family income to poverty ratio, education, physical exercise, BMI, smoking, drinking status, diabetes, hypertension, uACR, and eGFR.

### 3.6. Quantile-based g-computation (Q-gcomp) models

**Figure 4:**
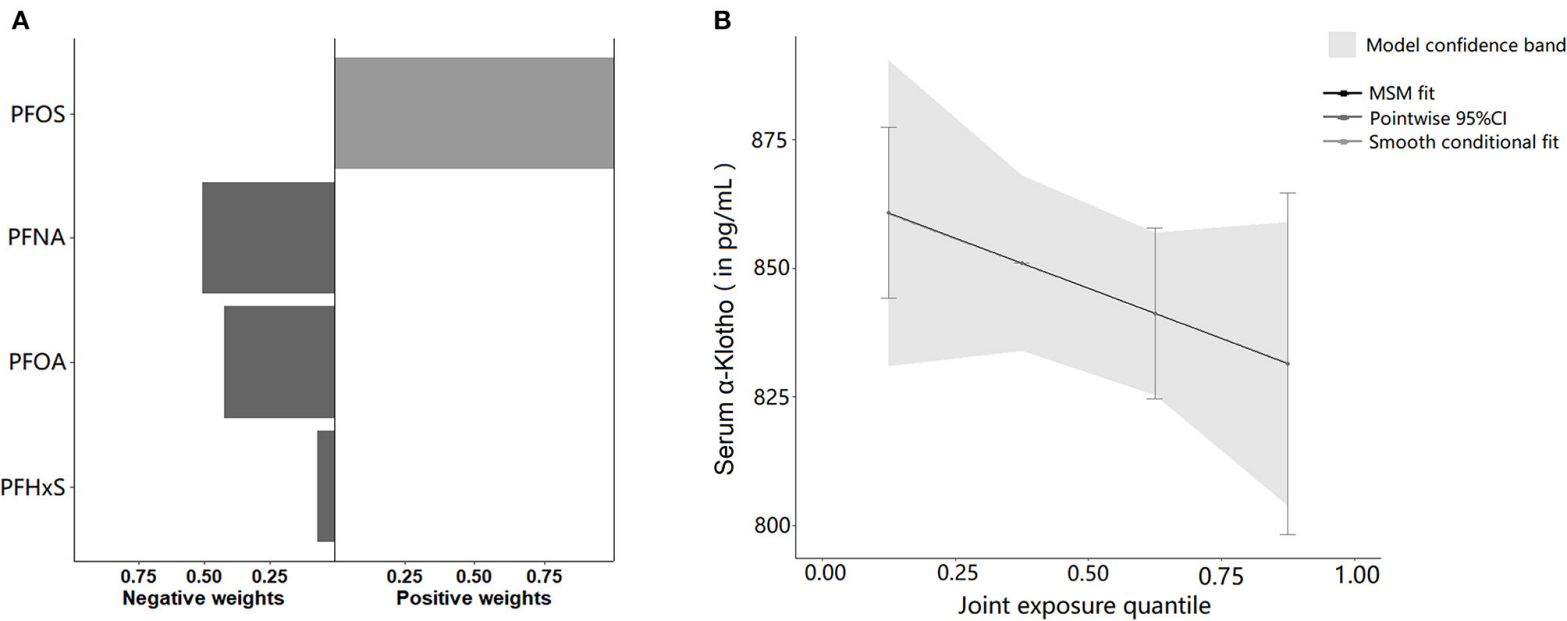
**(A)** The directions and magnitude of the assigned weights for each PFAS in relation to serum α-Klotho in quantile g-computation. **(B)** Mixed effects of four serum PFAS concentrations on serum α-Klotho. The model was adjusted for age, gender, race, family income to poverty ratio, education, physical activity, BMI, smoking, alcohol consumption, diabetes, hypertension, uACR, and eGFR.

After adjusting for all covariates, the mixture of the four PFAS showed a significant inverse relationship with α-Klotho concentration in the Q-gcomp analysis, PFNA was considered the major contributor and assigned the largest negative weight from Figure 4A. Furthermore, the estimated α-Klotho decreased linearly by quartile of PFAS mixture concentrations in Figure 4B.

## 4. Discussion

**Figure 5:**
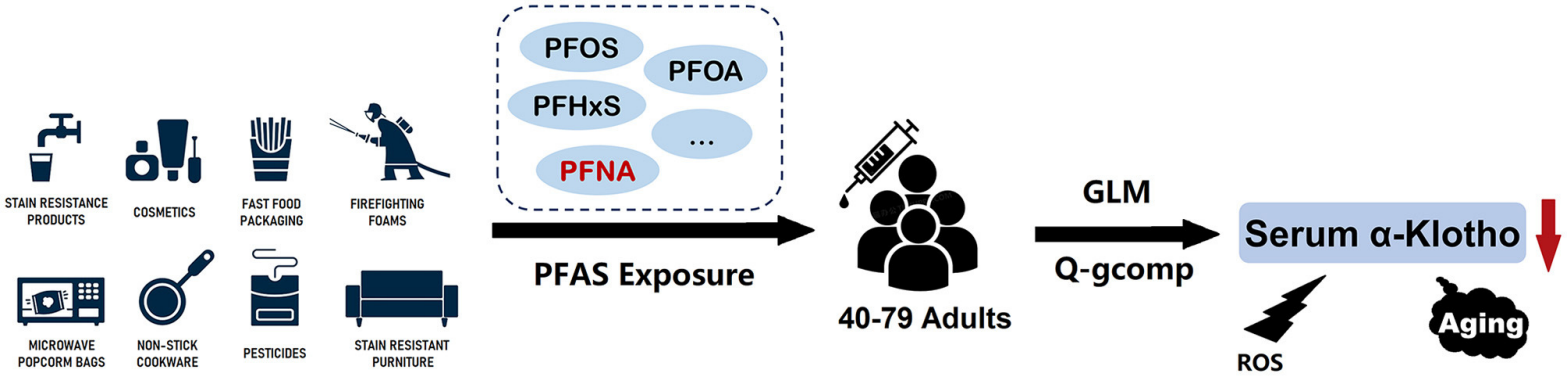
Overview of the results of the association between PFAS exposure and serum α-Klotho levels in middle-old aged participants.

Serum soluble α-Klotho, as an endocrine, plays an important role in regulating oxidative stress and the aging process. In this study, we investigated the association between four serum PFAS and serum α-Klotho levels in adults aged 40–79 years, using samples from the US. The main results were included in Figure 5. Interestingly, we found that PFAS exposure levels, specifically PFNA, were inversely associated with soluble α-Klotho serum contents in US adult participants after correcting potential confounding factors. The α-Klotho serum levels were decreased with increasing PFAS exposure levels, and this association was stronger in females aged from 40 to 59, providing new insights into the potential role of PFAS in oxidative stress and aging processes.

The variations in α-Klotho levels may be connected to the pathophysiology of the chronic kidney injury and neurotoxic effects associated with PFAS exposure according to previous studies (6, 36–38). Increasing studies have connected PFAS exposure to a rise in oxidative stress in the kidney, including DNA damage, increased production of mitochondrial transport chain proteins (39), decreased cell proliferation, and apoptosis (40). PFOS exposure might cause renal damage via multiple pathways, including dysregulation of PPARα and PPARγ, two key nuclear receptor hormones, highly expressed in the proximal renal tubule and involved in lipid metabolism, lipogenesis, glucose homeostasis, and cell growth and differentiation (41, 42). The renal tubular epithelial (KTE) cells exposed to PFOS showed a dramatic increase in apoptosis and fibrosis through Sirt1-mediated PPARγ deacetylation *in vitro* (43). An experimental study showed that maternal exposure to PFOS and PFNA resulted in reduced nephron counts and early hypertension in rat offspring (44). Accumulation of PFAS in the brain may have toxic effects on the central nervous system, including PFAS-induced behavioral and cognitive deficits, related with the effects of PFAS on calcium balance and alterations in neurotransmitters of neurons (45). Additionally, a-Klotho protein is a co-receptor of FGF23 and highly expressed in the kidney and brain. The kidney has the highest level of Klotho as a major source of soluble Klotho (46). Chronic kidney disease (CKD) has been a condition of Klotho deficiency, and animal models of CKD showed the reduced levels of total Klotho gene expression and in kidney tissue, with low soluble Klotho in circulation. In numerous experimental models, upregulating the soluble Klotho or the endogenous Klotho protected the kidney from injury, and inhibited renal fibrosis in CKD (47–49). In brain, Klotho is mainly produced by the choroid plexus and expressed at lower levels in specific brain regions, particularly in the hippocampus and pituitary (50). Behavioral studies on Klotho-knockout-mice showed deficits in memory retention compared with those of wild-type mice, possibly due to the increased oxidative stress in brain (51). In addition, Klotho may activate an antioxidant enzyme system, protecting hippocampus neurons against amyloid and glutamate toxicity, as a neuroprotective protein (52). Thus, decreased α-Klotho levels by PFAS exposure could explain some of the potential chronic kidney injury and neurotoxic effects from PFAS. However, further studies are needed to fully understand the regulatory role of α-Klotho on chronic kidney injury and neurotoxic effects after PFAS exposure.

It is still unclear how PFAS exposure regulates soluble α-Klotho levels in humans. The Klotho gene has been regulated by epigenetic and non-epigenetic mechanisms in renal disease (53). Exposure to higher levels of environmental pollutants (e.g., lead and cadmium) is associated with elevated levels of methylation of the Klotho gene, which may alter Klotho mRNA synthesis, leading to decreased protein levels of Klotho (54). Some PFAS, such as PFOA, PFNA, PFOS, and PFHxS had the potentials to cause oxidative stress in terms of ROS generation and DNA damage and were linked to indicators of inflammation and oxidative stress (11, 55). On the other hand, several studies on animals have revealed that higher Klotho was connected to lower oxidative stress (51, 56). PFAS exposure may affect human serum Klotho levels through multiple pathways. Future researches are currently needed to elucidate the specific biological processes or toxicological pathways by which PFAS exposure correlates inversely with human serum α-Klotho levels.

We performed multiple comparisons and observed age- and sex-specific associations in this study. Our study revealed higher serum concentrations of four PFAS in males than in females, consistent with the results of previous studies, and the possible reasons for these differences were mainly related to menstruation, pregnancy and placental transfer, breastfeeding, and other subtle causes, such as differences in serum albumin (57). The male data needed to be analyzed separately from the female data to obtain additional effects. The negative correlation between PFAS exposure and serum α-Klotho levels was the strongest in middle-aged females, suggesting that PFAS might have effects on endocrine and metabolic function and that women may be more sensitive to the deleterious effects of PFAS. The similar observations were consistent with other exposure studies in both animal and human studies (58, 59). A very comprehensive review of the relationship between PFAS exposure and liver injury by Costello et al. (60), showed that evidences from rodents consistently supported the results of human studies and suggested that PFAS exposure might lead to liver injury with elevated liver enzymes, steatosis, and histopathological changes. Female mice exhibited more lobular and portal inflammation compared to male mice exposed to PFAS (58). Analysis of population data also revealed gender differences between serum PFAS levels and liver function biomarkers, with PFOA and PFNA associated with clinically elevated alanine aminotransferase in female adolescents (59). Previous studies have found higher levels of soluble α-Klotho in patients with cirrhosis, suggesting that α-Klotho was also associated with impaired liver function (61). The age and sex-specific correlations of PFAS exposure with serum α-Klotho levels were observed in this study, as well as the hepatotoxic effects of PFAS in animal experiments and human studies, providing evidences for further investigations of the hepatotoxic toxic effects from PFAS exposure on age and sex in humans and the potential mediating role of α-Klotho.

Another interesting finding was that plasma levels of PFOS were lower in active smokers than those in non-smokers. Similar finding was made in a prior Danish cohort research of 1,076 pregnant women, which discovered that daily smoking during pregnancy was linked to lower maternal PFOS plasma levels than never smoking (62). A Danish cohort study of 652 men also reported finding higher plasma PFOS levels in non-smokers than in current smokers (63). These findings should reflect lifestyle differences between non-smokers and smokers related to PFOS exposure sources, or may reflect the increased elimination of PFOS by smokers. However, studies from three different geographic regions of Korea found the higher PFAS levels in smokers than in non-smokers (64), and several studies showed no significant association between serum PFOA levels and smoking (65, 66). Therefore, no consistent trends regarding smoking and PFAS exposure were observed in the studies.

Briefly, our results demonstrated a substantial correlation between low serum α-Klotho levels and blood PFAS, particularly PFNA. Numerous evidences suggested that PFAS was associated with ROS production and DNA methylation (11), while serum α-Klotho levels were regulated to some extent by ROS and DNA methylation (51). The causal pathway might be that PFAS in blood led to increased Klotho gene methylation or ROS production between PFAS exposure and serum α-Klotho levels, with decreased Klotho gene levels. Further studies are needed to confirm the related mechanisms.

Jain et al. firstly analyzed the association between α-Klotho concentrations and selected PFAS in US adults using NHANES data (24), and they found two important confounding factors, glomerular filtration rate status and albuminuria, and a modest correlation between PFAS exposure and low serum α-Klotho in healthy kidneys. Our study used correlation analysis and multiple general linear models to analyze the association between serum α-Klotho levels and four PFAS exposures with a detection rate higher than 98% in US adults, and also confirmed a negative association between PFAS exposure and serum α-Klotho levels, based on the NHANES database. In contrast to previous studies, we performed the subgroup analyses by age and sex further and used a mixed-methods approach to estimate the association between individual PFAS and mixtures of four PFAS with serum α-Klotho. This study again validated the negative association between PFAS exposure and serum Klotho, complementing the association characteristics of gender and age, and found that the mixture of the four PFAS also showed a significant inverse relationship with α-Klotho concentration, with PFNA as the main contributor. At the same time, we recognized that changes in urinary protein and renal function were gradual. So, we used the urine protein-creatinine ratio and eGFR for correction rather than the presence or absence of urine protein or SKD staging in order to better reflect the relationships.

Although the cross-sectional research design was unable to establish a causal connection, reverse causality was implausible given the long half-lives (several years) of the PFAS chemicals studied in this study. This investigation focused on the four most studied persistent PFAS chemicals, and further risk evaluation of other newer short-chain and alternative PFAS chemicals are needed to do urgently. Although we have included most of the potential confounders in this study, there are residual confounders. For instance, humans may be contemporaneously exposed to several environmental contaminants, such as heavy metals and p-dichlorobenzene (54, 67), possibly associated with serum α-Klotho levels.

## 5. Conclusion

In all, this study demonstrated that serum levels of PFAS, of which PFNA was a major contributor, may be inversely related to serum levels of α-Klotho in the US adult population. And the relationship was particularly strong in middle-aged females. This study may also explain the earlier reported chronic renal injury and neurotoxic effects associated with PFAS exposure, as circulating α-Klotho plays an important role in aging-related diseases such as renal system disorders and neurological diseases. Future researches are necessary to confirm the causal relationship between serum PFAS and α-Klotho levels and elucidate the possible intrinsic mechanisms.

## Data availability statement

Publicly available datasets were analyzed in this study. This data can be found at: https://www.cdc.gov/nchs/nhanes/index.htm.

## Ethics statement

Ethical review and approval was not required for the study on human participants in accordance with the local legislation and institutional requirements. Written informed consent for participation was not required for this study in accordance with the national legislation and the institutional requirements.

## Author contributions

ML and YM analyzed data and writing—original draft. WC, LZ, and CZ analyzed data and revised manuscript. WenjiZ and WenjuZ conceived the project, analyzed data, wrote the manuscript, provided supervision, and project administration. All authors were responsible for revising the manuscript, approved the final version, and read and agreed to the published version of the manuscript.
