# Supplementary material for: Association between perfluoroalkyl and polyfluoroalkyl internal exposure and serum α-Klotho levels in middle-old aged participants
Source: Front Public Health. 2023 May 4;11:1136454. doi: 10.3389/fpubh.2023.1136454 (PMC10204767; doi:10.3389/fpubh.2023.1136454)
Supplement: Supplementary file 1 [file Table_1.DOCX]

**Table S1. Distribution of serum per- and polyfluorinated substance concentrations (ng/mL) in adults aged 40 to 79 years in the NHANES 2013-2016 cycle.**

| Biomarkers | % Above LOD | GM (95%CI)  (ng/ml) | Percentile  (ng/ml) | | | LOD  (ng/ml) |
| --- | --- | --- | --- | --- | --- | --- |
|  |  |  | 25th | 50th | 75th |  |
| PFOA | 99.33 | 2.00(1.87, 2.14) | 1.27 | 1.97 | 2.97 | 0.10 |
| PFOS | 99.40 | 5.93(5.50, 6.39) | 3.80 | 6.30 | 10.6 | 0.10 |
| PFNA | 98.87 | 0.71(0.66, 0.77) | 0.50 | 0.80 | 1.20 | 0.10 |
| PFHxS | 98.73 | 1.41(1.29, 1.54) | 0.90 | 1.50 | 2.45 | 0.10 |

Note: GM, geometric means; LOD, limit of detection; PFOA, perfluorooctanoic acid; PFOS, perfluorooctane sulfonic acid; PFNA, perfluorononanoic acid; PFHxS, perfluorohexane sulfonic acid.
